# Electrophysiological Features of Atrial Flutter in Cardiac Sarcoidosis: A Report of Two Cases

**DOI:** 10.1016/s0972-6292(16)30568-x

**Published:** 2012-12-02

**Authors:** Narayanan Namboodiri, Martin K Stiles, Glenn D Young, Prashanthan Sanders

**Affiliations:** Centre for Heart Rhythm Disorders, University of Adelaide and Royal Adelaide Hospital, Adelaide, Australia

**Keywords:** atrial flutter, sarcoidosis, radiofrequency ablation

## Abstract

We report two cases of systemic sarcoidosis with atrial flutter as the clinical manifestation. In one patient, who had symptoms of shorter duration, the arrhythmia was no longer inducible after a course of glucocorticoid therapy. Electroanatomical mapping in the other case revealed patchy fibrosis of the left atrial myocardium and multiple macro-reentrant circuits. Sinus rhythm could be restored with ablation of these reentrant circuits. To our knowledge, this is the first report on the demonstration of atrial scarring in a patient with sarcoidosis using 3-D electroanatomical mapping. These two cases illustrate that the inflammation of atrial myocardium is the primary mechanism of atrial arrhythmias in patients with cardiac sarcoidosis.

## Introduction

Sarcoidosis is a systemic granulomatous disease of unknown etiology, with clinical cardiac involvement varying from 20 to 60% of patients [1,21], most often manifesting as abnormalities in the conduction or with pericardial involvement. Atrial arrhythmia or granulomatous involvement of the atrial myocardium has rarely been described in them [2,3]. Here we report 2 patients who presented with atrial arrhythmias as the isolated cardiac manifestation of sarcoidosis. Based on the findings on electrophysiological (EP) study including 3-D electroanaomical mapping, we propose evidence for direct involvement of the atrial myocardium in the form of inflammation and scarring in cardiac sarcoidosis, which could guide the therapeutic approach in these patients.

## Case 1

**Figure 1:**
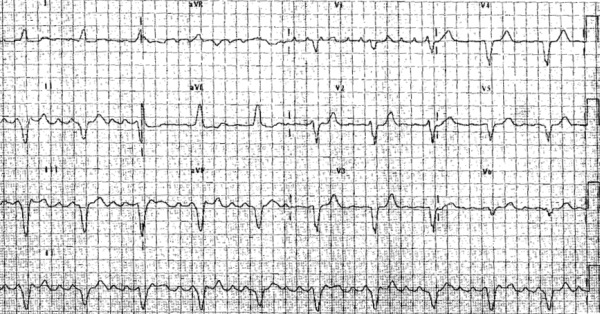
ECG of flutter in case 1. The morphology of flutter waves suggests a left atrial origin. Note the intraventricular conduction defect also

A 49-year-old male, with a permanent pacemaker implanted for complete heart block and history of iritis a few months before, presented with recurrent episodes of palpitation over the preceding few weeks. The 12-lead electrocardiogram was suggestive of atypical atrial flutter of probable left atrial origin (Figure 1). The echocardiogram was normal except for mildly dilated atria. There was no history of hypertension, diabetes or dyslipidemia. A computerized tomographic (CT) scan of the thorax done to define atrial anatomy prior to ablation showed multiple nodules in the lung parenchyma, which on transbronchial lung biopsy was suggestive of sarcoidosis. An elective EP study was planned, and in the meantime he was treated with prednisolone 1mg/kg/day and sotalol 40 mg/day. Atrial flutter could not be induced during EP study performed after 2 months of steroid therapy. He remained asymptomatic thereafter, and device interrogation till 24 months after EP study revealed only a few episodes of non-sustained atrial tachycardia.

## Case 2

**Figure 2:**
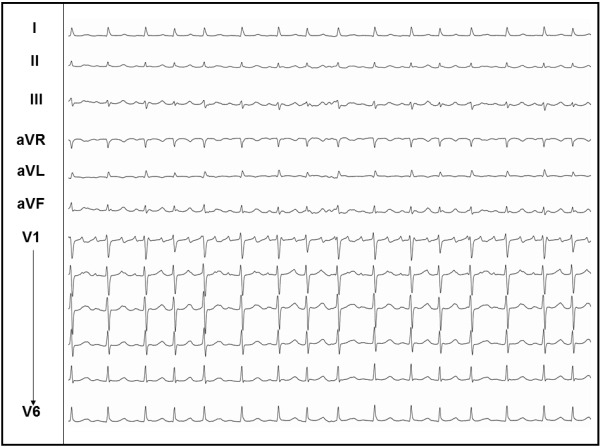
ECG of flutter in case 2

**Figure 3:**
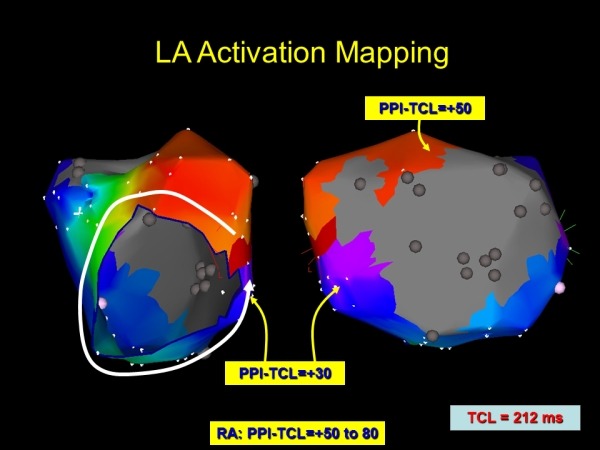
Electroanatomic map of the left atrium during flutter presented in Figure 2. A large area of scar is noted along the posterior left atrium. Activation mapping demonstrates a counter-clockwise peri-mitral macro-reentry. Entrainment demonstrates the best post pacing interval (PPI) at the lateral mitral isthmus

**Figure 4:**
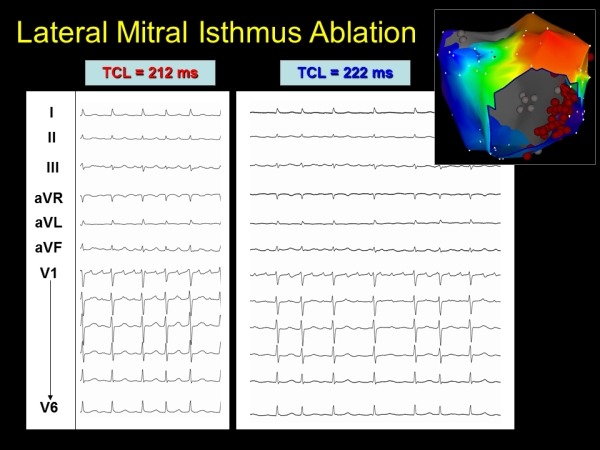
Subtle change in morphology and tachycardia cycle length (TCL) with lateral mitral isthmus ablation

**Figure 5:**
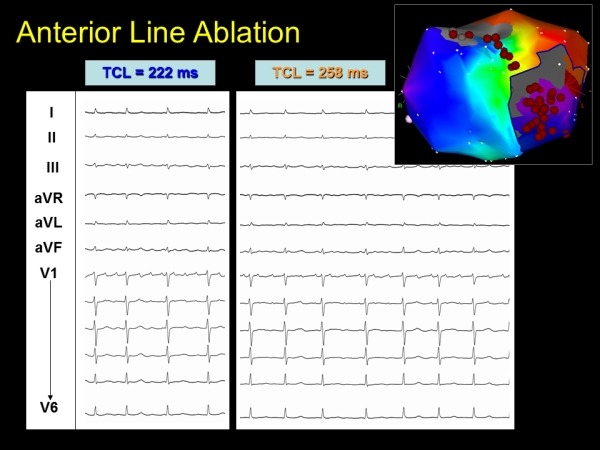
Further change in tachycardia cycle length (TCL) and morphology with anterior mitral isthmus line

**Figure 6:**
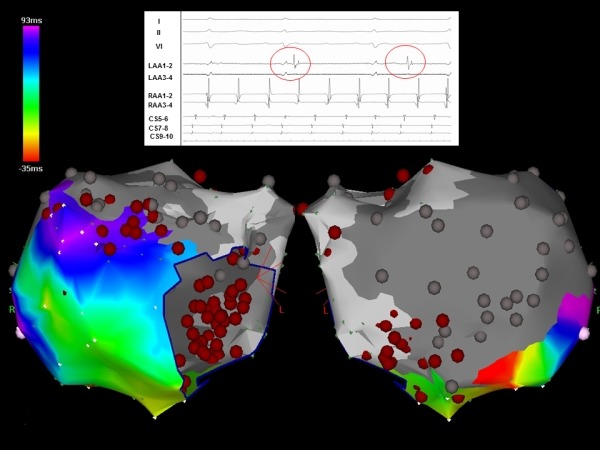
Inadvertent isolation of the entire lateral left atrium. Note the electrogram insert shows dissociated activity (red circles) within the left atrium

**Figure 7:**
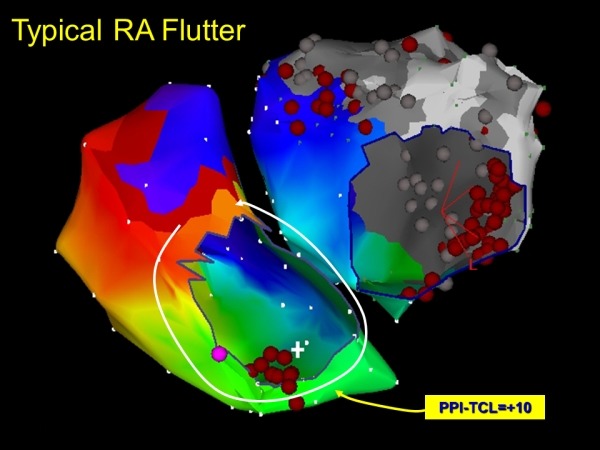
Repeat mapping demonstrates typical cavotricuspid isthmus dependent macro-reentry

A 48-year-old woman was referred to us with recurrent episodes of paroxysmal atrial flutter, treated with a failed attempt at ablation and pharmacotherapy (flecainide, sotalol and atenolol) in the past. Chest X-ray followed by CT scan of the thorax suggested type III pulmonary sarcoidosis with extensive involvement of the lung parenchyma. Echocardiography showed a mildly dilated left ventricle (LV) and left atrium (LA, anteroposterior diameter of 45 mm) with normal LV systolic function. Electrocardiogram suggested LA flutter with varying atrioventricular conduction (Figure 2). After treatment with prednisolone (1 mg/kg/day) for 6 weeks, she was taken for EP study. Atrial flutter (cycle length of 212 ms) could not be entrained from right atrium, with postpacing interval (PPI) exceeding tachycardia cycle length (TCL) by more than 50 ms. Voltage mapping of the LA performed with CARTO system (Biosense-Webster), with scar defined as the absence of recordable activity or a bipolar voltage amplitude ≤ 0.05 mV, suggested extensive scarring of the posterior LA wall (Figure 3). The tachycardia could be entrained from lateral mitral isthmus (PPI - TCL = 30 ms). Ablation of the lateral mitral isthmus was performed using an externally irrigated catheter (Thermocool, Biosense-Webster, 35 W), resulted in continuation of flutter with prolongation of TCL by 10ms (Figure 4). Assuming incomplete block, an anterior mitral isthmus line was attempted joining the anterior scar to the mitral annulus (Figure 5). This resulted in an abrupt prolongation of the TCL to 258 ms (Figure 6). Repeat mapping of the atria demonstrated dissociated activity in the LA appendage confirmed the complete block at both these ablation lines with inadvertent isolation of the lateral left atrium (Figure 6). The slower flutter was confirmed to be cavotricuspid isthmus dependent (Figure 7; PPI - TCL = 10 msec). The flutter terminated on ablation of cavotricuspid isthmus. The endpoint was bidirectional block on established criteria. The patient has remained in sinus rhythm after 30 months of follow up.

## Discussion

The tissue changes in sarcoidosis are characterized by accumulation of T lymphocytes and mononuclear phagocytes, non-caseating epithelioid granulomas, and derangements of normal tissue architecture, all leading to parenchymal damage and irreversible tissue fibrosis [3]. Granulomatous lesions in sarcoidosis are known to occur in endocardium, pericardium and ventricular myocardium, the latter being the most common site. In a necropsy study in 113 patients with cardiac sarcoidosis, Roberts et al noted extensive ventricular scarring as a result of healing of granulomas in all [4]. However, only 2 of them had granulomas in atrial walls. Atrial arrhythmia as the sole cardiac clinical manifestation of sarcoidosis has not been described thus far in the literature.

The development of atrial or ventricular arrhythmias can result in significant hemodynamic and clinical deterioration in these patients with a high incidence of co-existing systolic or diastolic dysfunction. The mechanism for ventricular tachyarrhythmias is thought to be mediated via granulomas and myocardial fibrosis, while atrioventricular block occurs presumably secondary to nodal artery ischemia or granulomas in the basal septum. Atrial arrhythmia in sarcoidosis, a less well-characterized entity, was proposed to be more due to atrial dilatation secondary to ventricular, valvular or pulmonary involvement rather than direct involvement of the atrial myocardium [3]. Our patients had neither the involvement of these structures on echocardiography nor any systemic risk factors known to result in atrial arrhythmias. In addition, the response to anti-inflammatory drugs in the early stage of cardiac involvement in the first case and the demonstration of extensive scarring of atrial wall in the second, clearly suggest a stage of active inflammation followed by the development of fibrosis and scarring of atrial myocardium in the natural history of cardiac sarcoidosis. We could not have a histological evidence to support this as the biopsy of atrial myocardium was not attempted due to ethical and safety reasons.

To our knowledge, this is the first report on the demonstration of atrial scarring in a patient with sarcoidosis by voltage mapping using any 3-D electroanatomical mapping system. Our index patient had no other risk factors which could otherwise account for the scarring of the LA myocardium. The EP findings in this case suggest that the atrial myocardium can be directly involved in sarcoidosis resulting in patchy areas of scarring interspersed with conducting atrial myocardium, an ideal substrate for multiple macro-reentrant circuits. Recognition and ablation of these circuits can result in long-term maintenance of sinus rhythm in these patients.
